# ClpAP protease is a universal factor that activates the *parDE* toxin-antitoxin system from a broad host range RK2 plasmid

**DOI:** 10.1038/s41598-018-33726-y

**Published:** 2018-10-16

**Authors:** Andrzej Dubiel, Katarzyna Wegrzyn, Adam P. Kupinski, Igor Konieczny

**Affiliations:** 1 Department of Molecular and Cellular Biology, Intercollegiate Faculty of Biotechnology, University of Gdansk and Medical University of Gdansk, Abrahama 58, 80-307 Gdansk, Poland; 2 Present Address: Ipsen Bioinnovation, 102 Park Drive, Milton Park, Abingdon, Oxfordshire OX14 4RY UK

## Abstract

The activity of type II toxin-antitoxin systems (TA), which are responsible for many important features of bacterial cells, is based on the differences between toxin and antitoxin stabilities. The antitoxin lability results from bacterial protease activity. Here, we investigated how particular *Escherichia coli* cytosolic proteases, namely, Lon, ClpAP, ClpXP, and ClpYQ, affect the stability of both the toxin and antitoxin components of the *parDE* system from the broad host range plasmid RK2. The results of our *in vivo* and *in vitro* experiments show that the ParD antitoxin is degraded by the ClpAP protease, and dsDNA stimulates this process. The ParE toxin is not degraded by any of these proteases and can therefore cause growth inhibition of plasmid-free cells after an unequal plasmid distribution during cell division. We also demonstrate that the ParE toxin interaction with ParD prevents antitoxin proteolysis by ClpAP; however, this interaction does not prevent the ClpAP interaction with ParD. We show that ClpAP protease homologs affect plasmid stability in other bacterial species, indicating that ClpAP is a universal activator of the *parDE* system and that ParD is a universal substrate for ClpAP.

## Introduction

Toxin-antitoxin systems (TA) are widely distributed among prokaryotes. Until now, homologous systems in eukaryotes have not been identified^1^. TA system components may be encoded by bacterial chromosomes or plasmids. Depending on their location the functions might vary. The role of some chromosomal TA systems is still unclear, but they are mainly responsible for the response to environmental stress^2–4^ and are involved in the formation of persister cells during stress conditions^5–7^. Chromosomal TA modules have also been correlated to bacterial infections^8,9^. The main function of plasmidic TA systems is to maintain plasmids in host cell populations without any selection pressure. It was proposed that plasmidic TA induces post-segregational killing (psk) in cells lacking plasmids after unequal plasmid distribution during cell division^10,11^. However, no direct evidence of cell killing of plasmid-free segregates has been demonstrated, and it is not clear if the daughter cells lacking the plasmid are killed or just outcompeted due to their slow growth^12^. Literature reports demonstrate that plasmidic TA systems can also contribute to effects comparable to the chromosomal TA systems in host cells and affect the response to environmental stress. The *ccdAB* TA system from the F plasmid affects persister cell formation and protects against cell death under antibiotic stress conditions^13,14^. Plasmid TA modules can also result in host strain virulence. The *vapBC* TA module from the plasmid pSLT confers *Salmonella* Typhimurium virulence and stabilizes the virulence plasmid of this species^15,16^. These observations make plasmidic TA systems more intriguing, since they not only provide basic maintenance functions of the plasmid DNA but the host cell may also benefit under stress conditions.

Six types of TA systems are currently distinguishable on the basis of the form and the exact action of the antitoxin that protects a cell from toxicity^17^. In most cases, the toxin is a very stable protein whose activity may cause reversible bacterial metabolic dormancy (bacteriostasis) or even cell death. Type II antitoxins are small unstable proteins that are typically composed of two functional regions: an N-terminal DNA-binding domain (DBD) and C-terminal region involved in toxin binding^18–20^. Formation of the toxin-antitoxin complex results in inhibition of toxin activity towards a cellular target. These complexes are also often responsible for the autoregulation of the TA operon^21,22^. In TA type II systems, the factor that activates the system is a protease that is responsible for degradation of the antitoxin. Type II TA systems were originally found in low copy number plasmids (e.g., RK2).

RK2 is a 60 kbp broad host range plasmid that has the ability to replicate and be stably maintained in many distantly related species of bacteria^23,24^. Research on RK2 has shown that in addition to genes that ensure the accurate performance of processes such as replication initiation, expression regulation of the *rep* gene, formation of a handcuff complex, plasmid multimer resolution (mrs), and partitioning of plasmid particles into cells before cell division^17,25,26^, RK2 also has a *parDE* operon that allows its efficient maintenance in host cells^27^. Further studies have confirmed that this operon codes for genes of the type II toxin-antitoxin system, where ParD is an antitoxin and ParE is a toxin^26,28,29^.

Native ParD from RK2 is a homodimer^29,30^ that exhibits high thermal stability and excellent refolding properties after heat-induced denaturation^31,32^. ParD is composed of α-helical and β-strand regions. It consists of two structurally distinct moieties: a well-ordered N-terminus and an unstructured C-terminus^33^. Native RK2 ParE protein forms a homodimer^29^. It is homologous with YoeB and RelE toxins from *Escherichia coli* and ParE protein from *Caulobacter crescentus*. The crystal structure of *C*. *crescentus* ParE reveals that it contains two antiparallel α-helices at the N-terminus that form a hairpin and pack against a three-stranded antiparallel β-sheet^34^. Although RK2 ParE is highly homologous to the RelE toxin at the level of primary sequence and tertiary structure, it does not contain any of the three critical catalytic residues required for mRNA cleavage at the ribosome^35^. The cellular target of RK2 ParE toxin is a DNA gyrase. ParE alters gyrase activity, which results in DNA nicking and the formation of improper linear forms of chromosomal DNA^36^. As yet, the protease responsible for degrading the ParD antitoxin of the *parDE* system has not been identified.

In *E*. *coli*, four cytosolic AAA+ proteases have been identified: ClpXP, ClpAP, ClpYQ (also referred to as HslUV) and Lon^37^. The AAA+ proteases are invariably composed of an ATP-dependent unfoldase, which is a molecular chaperone belonging to the AAA+ (ATPase associated with various cellular activities) protein superfamily that is responsible for substrate recognition and processing, and a peptidase that forms the proteolytic chamber^37,38^. The recognition of a substrate protein is based either on detection of hydrophobic stretches within the substrate or by binding to specific motifs called degrons^39,40^. Proteases may also interact with adaptor proteins that help to deliver a substrate to the protease^40,41^.

In this study, we aimed to unravel the activation of the *parDE* toxin-antitoxin system from the RK2 plasmid by analyzing the ParD and ParE protein stabilities. To identify the potential protease(s) responsible for the degradation of *parDE* protein system components, we performed *in vitro* proteolytic assays using Lon, ClpAP, ClpXP and ClpYQ proteases, SPR analysis of protein-protein interactions and *in vivo* stability assays using wild-type and protease-deficient strains of *E*. *coli*, *C*. *crescentus* and *Pseudomonas putida*.

## Results

### ClpAP protease is responsible for ParD protein degradation

**Figure 1:**
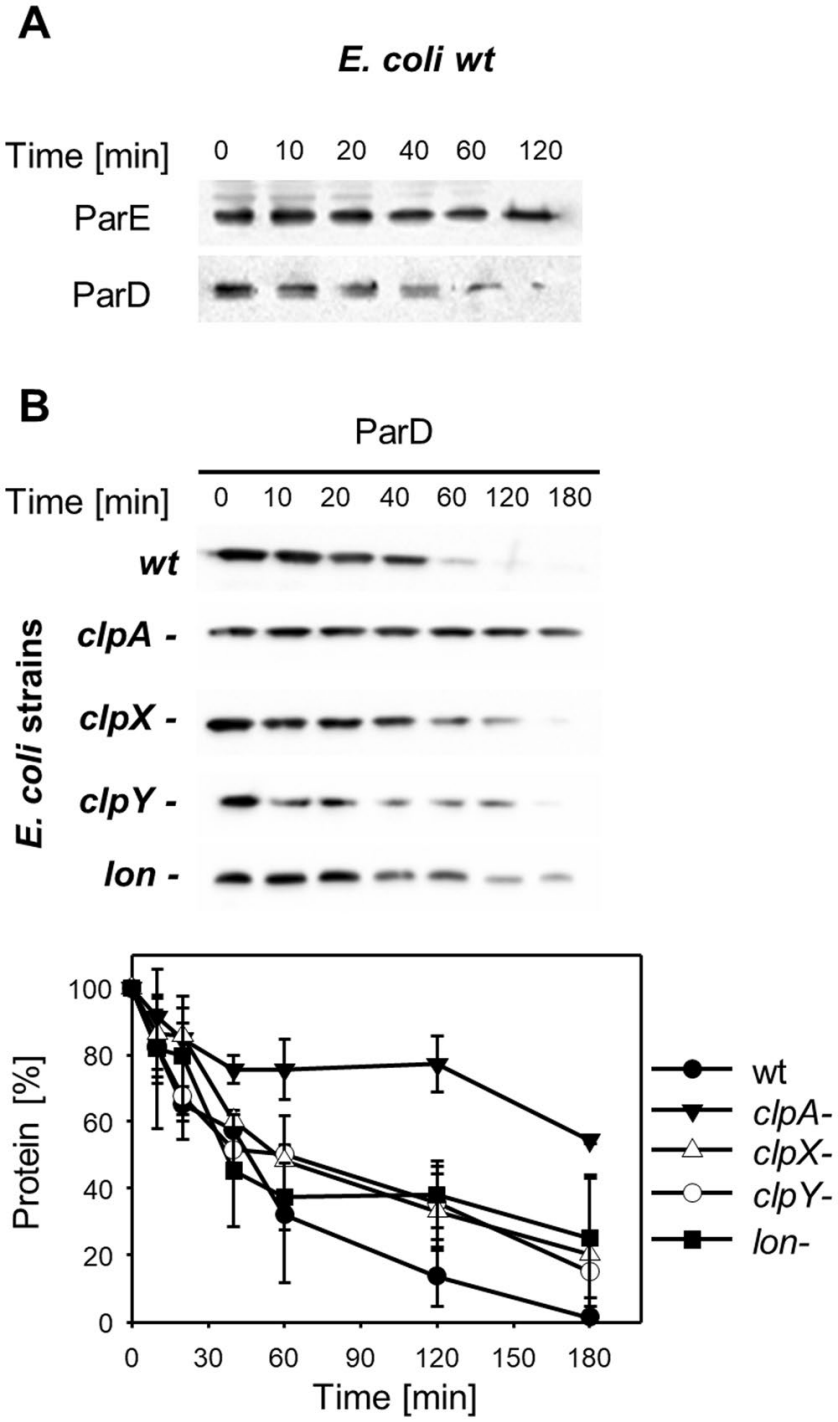
The stability of ParD antitoxin is substantially increased in the *E*. *coli clpA-*deficient strain. (**A**) In the wild-type *E*. *coli* strain, the stability of ParD and ParE proteins was analyzed after inhibition of translation by tetracycline of ParD- (pBAD24-ParD) and ParE- (pRR46 and pAS12) overproducing cells. The assay was performed as described in Methods. (**B**) Cells of *E*. *coli wt* (●), *clpA*(−) (▼), *clpX*(−) (Δ), *clpY*(−) (○), and *lon*(−) (■) harboring plasmid pBAD24-ParD for overproduction of ParD were used. Samples were taken from the cultures at the indicated time points after the addition of tetracycline and were analyzed for ParD presence. The assay was performed as described in Methods. Full-length blots are included in the Supplementary Information file (Fig. S11). Each experiment was repeated three times, and the mean values with standard deviations (error bars) are presented as graphs. Numeric data and P value are shown in the Supplementary Table S1.

Since the stability of ParD and the potential protease responsible for its degradation have not been clarified, we conducted ParD stability tests. First, the stability of the ParD protein was verified *in vivo* in *E*. *coli* C600 cells. The plasmid pBAD24_ParD was used, which allows the arabinose-dependent expression of *parD*. One hour after induction, the protein translation was inhibited by the addition of tetracycline. Samples were collected at selected time points. After 120 min, a significant protein loss was observed by Western blot analysis with anti-ParD antibodies (Fig. 1A). *E*. *coli* strains with protease gene inactivation, *lon(−)*, or AAA+ protease subunits *clpA*(−), *clpX*(−), and *clpY*(−) were also used for the analysis of ParD stability. We observed a significant (P value 1.3 × 10^−3^)(see Supplementary Table S1) increase in the stability of the ParD protein in the *clpA*(−) strain (Fig. 1B). This result indicates that the ClpAP protease is responsible for the efficient proteolysis of ParD.

We also analyzed whether the ParD protein contained known ClpA substrate recognition motifs. The ParD sequence analysis revealed that, indeed, ParD contains two putative motifs for ClpA recognition (see Supplementary Fig. S1). One motif is located in the protein C-terminal part and the other one, which is SsrA-like, is located closer to the N-terminus.

### ParE protein is stable in host cells

The stability of the ParE protein was also tested. *In vivo* analysis showed that the ParE protein was stable after 120 min (Fig. 1A). ParE stability was also analyzed using *in vitro* proteolysis experiments with Lon, ClpAP, ClpXP, and ClpYQ proteases (see Supplementary Fig. S2). No degradation was observed after 120 min.

### DNA stimulates ClpAP protease to degrade ParD

**Figure 2:**
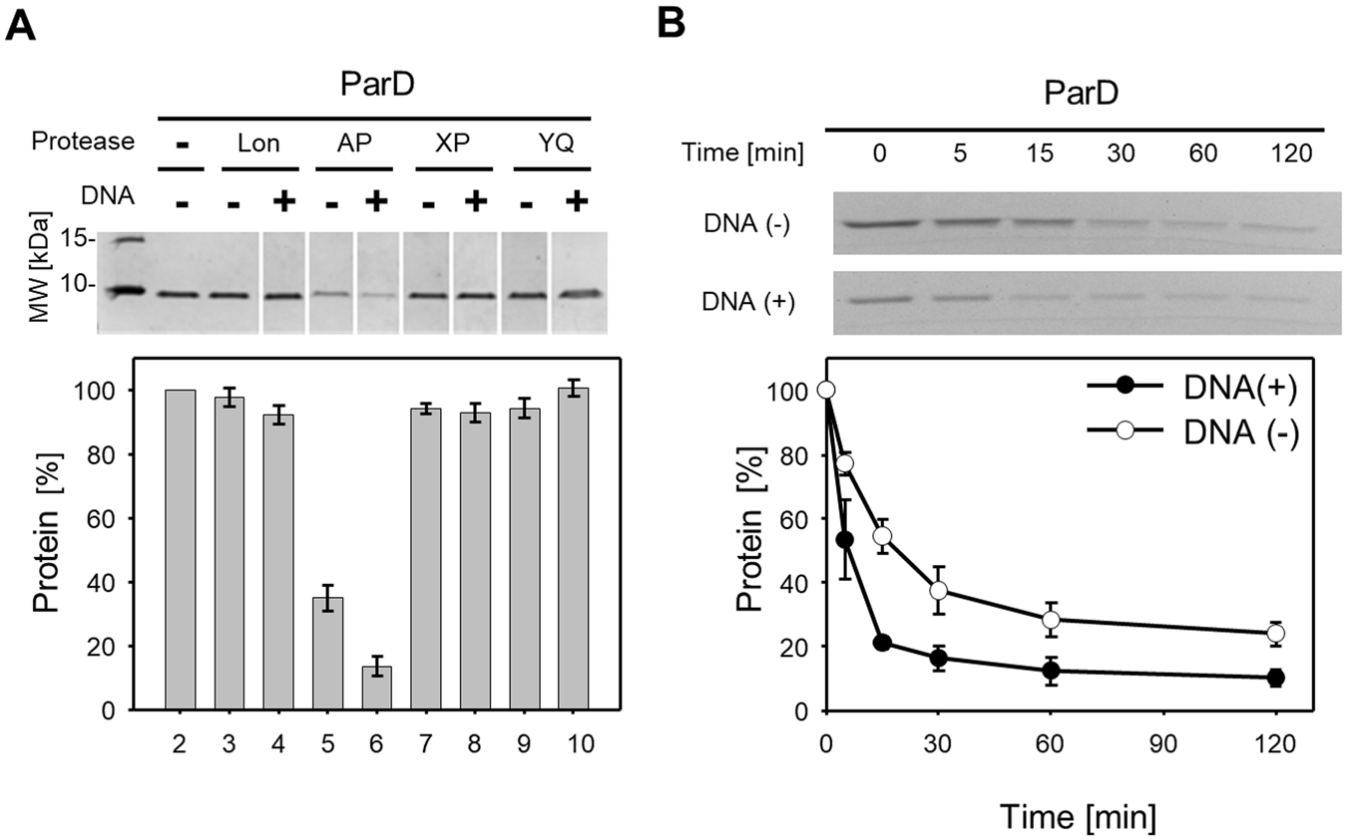
ParD is degraded by *E*. *coli* ClpAP protease. (**A**) *In vitro* proteolysis assay of the analysis of degradation of ParD antitoxin by *E*. *coli* proteases Lon (lane 3 and 4), ClpAP (lane 5 and 6), ClpXP (lane 7 and 8), and ClpYQ (lane 9 and 10). Supercoiled pKD19L plasmid (300 ng) was added to the reaction mixtures (lane 4, 6, 8 and 10). Negative control, no protease added (lane 2). Molecular weight marker (lane 1). The experiment was carried out for 120 min. (**B**) Comparison of ParD degradation rate by ClpAP in the presence (●) or absence (○) of DNA. Mixtures containing ClpAP with or without DNA were initially preincubated for 5 min, then the ParD was added. Reactions were stopped at the indicated time points by the addition of 8 μl of 4x Laemmli buffer. The assay was performed as described in Methods. Full-length gels are included in the Supplementary Information file (Fig. S12). Each experiment was repeated three times, and the mean values with standard deviations (error bars) are presented as graphs. Numeric data and P value are shown in the Table S2.

The ClpA unit of the ClpAP protease is responsible for recognition of a substrate, ATP-dependent substrate unfolding, and its translocation into the central channel of the ClpP proteolytic subunit^42,43^. Previous results have shown that the ClpAP protease is able to bind DNA. Moreover, ClpAP-DNA interaction increases its ATPase activity and the efficiency of substrate proteolysis^44^. We tested whether DNA affects ParD proteolysis. *In vitro* experiments were performed using the Lon, ClpAP, ClpXP and ClpYQ proteases. A significant decrease in the amount of ParD protein (using SDS-PAGE analysis) was observed when ClpAP protease was in the reaction mixture (Fig. 2A). We also noticed that the presence of supercoiled plasmid DNA increased the efficiency of the proteolysis (Fig. 2A). To further analyze the influence of DNA on ParD degradation, various DNA variants were used containing or lacking the promotor sequence *pparDE* (ParD protein binds a site in the *parDE* operon promoter^30^) and two DNA forms: supercoiled or linear (see Supplementary Fig. S3). In all cases a significant increase in proteolysis was observed. This suggests that ParD is efficiently processed by ClpAP in the presence of DNA regardless of the *pparDE* sequence or DNA form. To determine the dynamics of ParD degradation by ClpAP, a time-course *in vitro* proteolysis assay was performed (Fig. 2B). The analysis showed that approximately 50% of the antitoxin level decreased in less than 5 min. in the presence of DNA. The experiment was also performed in the absence of DNA, and the result showed that the time required to reach fifty percent was tripled. For statistical data analysis see also Supplementary Table S2. These results suggest that ParD degradation is rapid and is stimulated by DNA.

**Figure 3:**
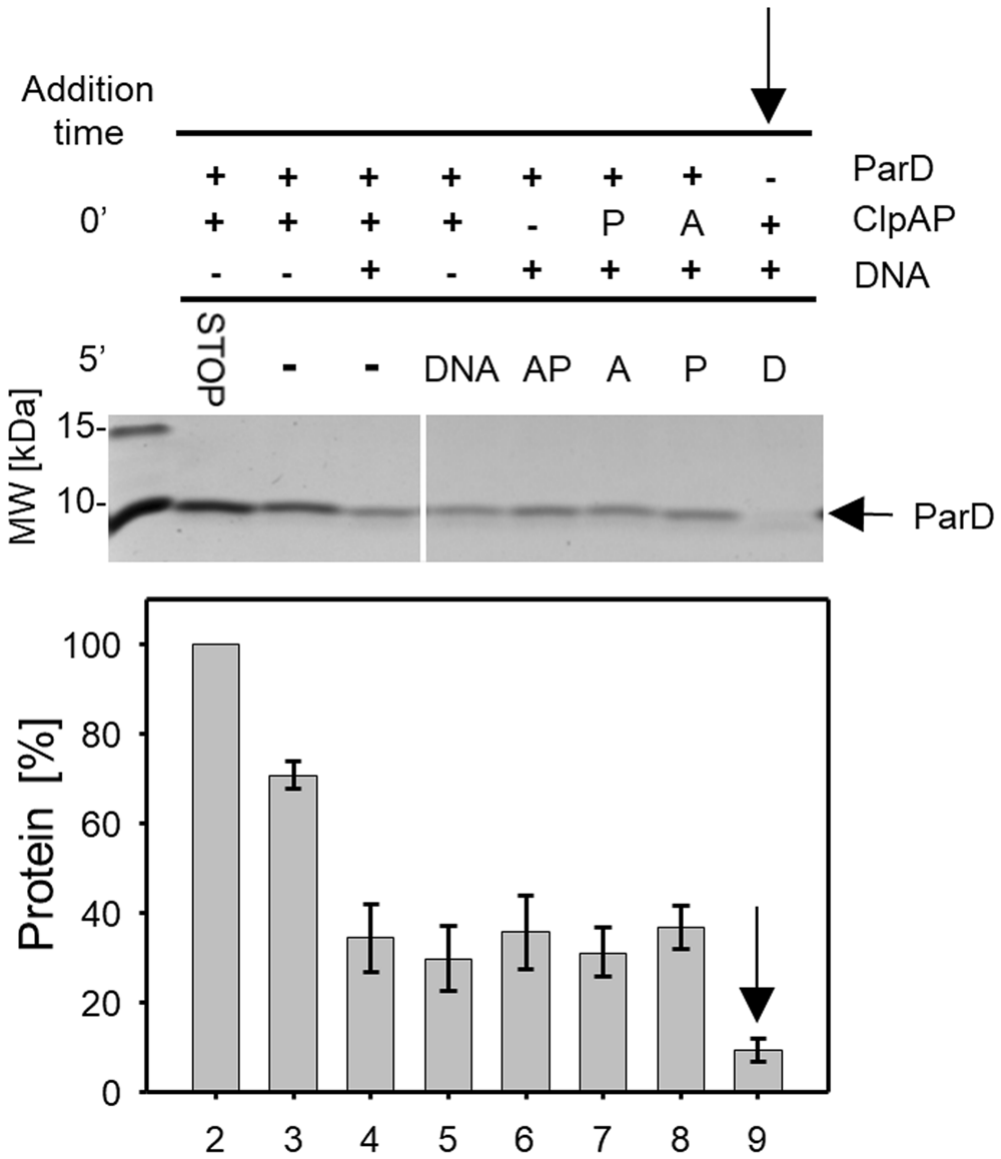
Addition order of reaction components affects the efficiency of ParD degradation by ClpAP *in vitro*. Reaction components ParD (marked as D), pKD19L (marked as DNA) and protease complex subunits ClpA (marked as A) and ClpP (marked as P) were mixed together in different orders. Mixtures containing two or three components were initially preincubated for 5 min, then the remaining components were added and the reaction continued for 30 min (lane 5–9). After a 5 min pre-incubation step, no components were added and the mixtures were incubated for 30 min (lane 3 and 4). The control reaction was stopped after 5 min of preincubation (lane 2). Molecular weight marker (lane 1). The most efficient degradation reaction is marked with an arrow (**↓**). The assay was performed as described in Methods. Full-length gels are included in the Supplementary Information file (Fig. S13). Each experiment was repeated three times, and the mean values with standard deviations (error bars) are presented as graphs.

To test whether and how the preformation of complexes (protease, DNA, and/or ParD) affect the processing of ParD by ClpAP, we performed an experiment in which the addition order of proteolytic components was changed. In each case, DNA stimulated the degradation of the substrate; however, when the ClpAP protease was preincubated with DNA before the substrate ParD was added, the stimulation was the highest (Fig. 3). This sequential assembling of the proteolytic complex suggests that the ClpAP protease is stimulated by DNA to process the substrate.

We also tested whether DNA could stimulate ParE degradation. The presence of DNA in the reaction mixture did not result in ParE proteolysis *in vitro* by Lon, ClpAP, ClpXP or ClpYQ proteases (see Supplementary Fig. S2).

### ParD stability increases when it is bound to ParE

**Figure 4:**
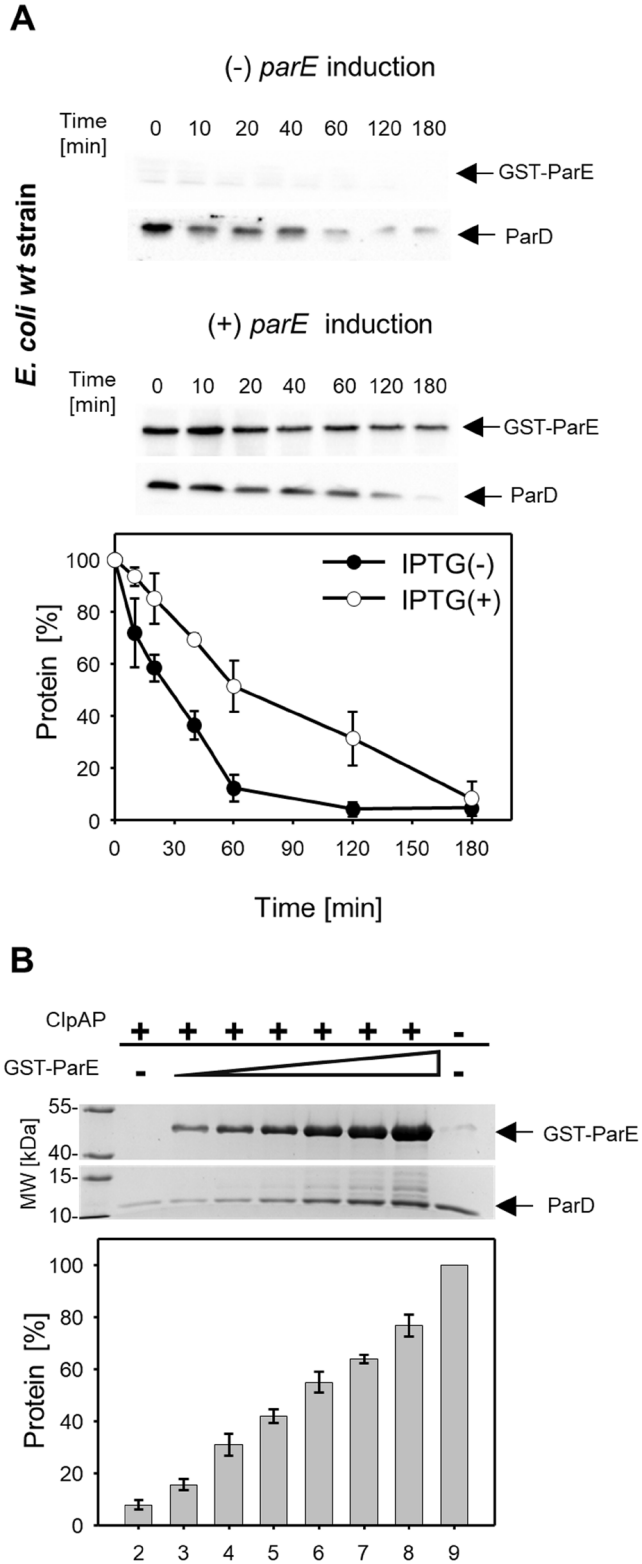
ParE protects ParD from proteolysis by ClpAP. (**A**) *In vivo* stability of ParD in a wild-type strain harboring plasmids for the production of ParD (constitutive) and over-production of GST-ParE (IPTG inducible) in the absence (●) or presence (○) of ParE. The assay was performed as described in Methods. Samples taken from the cultures at the indicated time points were analyzed for ParD and ParE presence by SDS–PAGE, followed by immunoblot with anti-ParD and anti-ParE antibodies. Each experiment was repeated three times, and the mean values with standard deviations (error bars) are presented as graphs. Numeric data and P value are shown in the Supplementary Table S3. (**B**) *In vitro* proteolysis assay of ParD antitoxin (1.5 µg) by ClpAP protease in the presence of ParE. Increasing concentrations of GST-ParE were added to the reaction mixtures (lanes 3–8: 0.2, 0.4, 0.75, 1.5, 3 and 6 µg, respectively). In a positive control reaction, no GST-ParE was added (lane 2). In a negative control reaction, no protease was added (lane 8). Molecular weight marker (lane 1). The experiment was carried out for 120 min. The assay was performed as described in Methods and analyzed with SDS–PAGE, followed by Coomassie brilliant blue staining. Given values are means from three independent repeats of each experiment. Full-length blots and gels are included in the Supplementary Information file (Fig. S14).

To investigate the effect of the ParE protein on the proteolysis of the ParD protein by the ClpAP protease, we performed a series of *in vivo* and *in vitro* experiments. The *E*. *coli* C600 strain was transformed with plasmid pAS12, which encodes the *GST-parE* gene (expression induced by IPTG), and the pRR46 plasmid for the constitutive expression of *parD*. Overnight cultures were diluted 1:100 in fresh LB broth, and two parallel cultures were grown at 37 °C to an OD_600_ of 0.6. At this point, expression of ParE was induced in one culture, and the second culture was without IPTG induction of *parE* gene expression. One hour after the induction, the protein translation was inhibited by the addition of tetracycline. Samples were collected at selected time points and analyzed as described in Methods. The analysis was carried out using anti-ParD and anti-ParE antibodies. When the ParE toxin was overproduced in host cells, the stability of the ParD antitoxin increased (Fig. 4A, statistical analysis Supplementary Table S3). Taking into account the size of the ParD and ParE proteins and the sensitivity of the antibodies used, the estimated ParD:ParE stoichiometry in the reaction after tetracycline addition (time 0) was 1:1, indicating that ParD-ParE could assembled as a heterotetramer. In the *E*. *coli clpA* (−) strain, we did not observe any significant differences in the stability of the ParD antitoxin regardless of induction of *parE* expression (see Supplementary Fig. S4). In the *in vitro* proteolysis assay, an increasing concentration of ParE protein was used in the reaction mixtures containing ParD and ClpAP. SDS–PAGE followed by Coomassie brilliant blue staining showed that proteolysis of the ParD protein by the ClpAP protease was less efficient with increasing amounts of ParE (Fig. 4B). The estimated ParD:ParE stoichiometry of the reactions was 1:0.03 up to 1:1 and was comparable to that obtained in the *in vivo* tests (see Fig. 4A). The ParE toxin had no effect on the proteolysis of the other substrates by ClpAP protease (see Supplementary Fig. S5). In a control experiment we also showed that the GST-tag had no effect on ParD proteolysis by ClpAP (see Supplementary Fig. S6).

**Figure 5:**
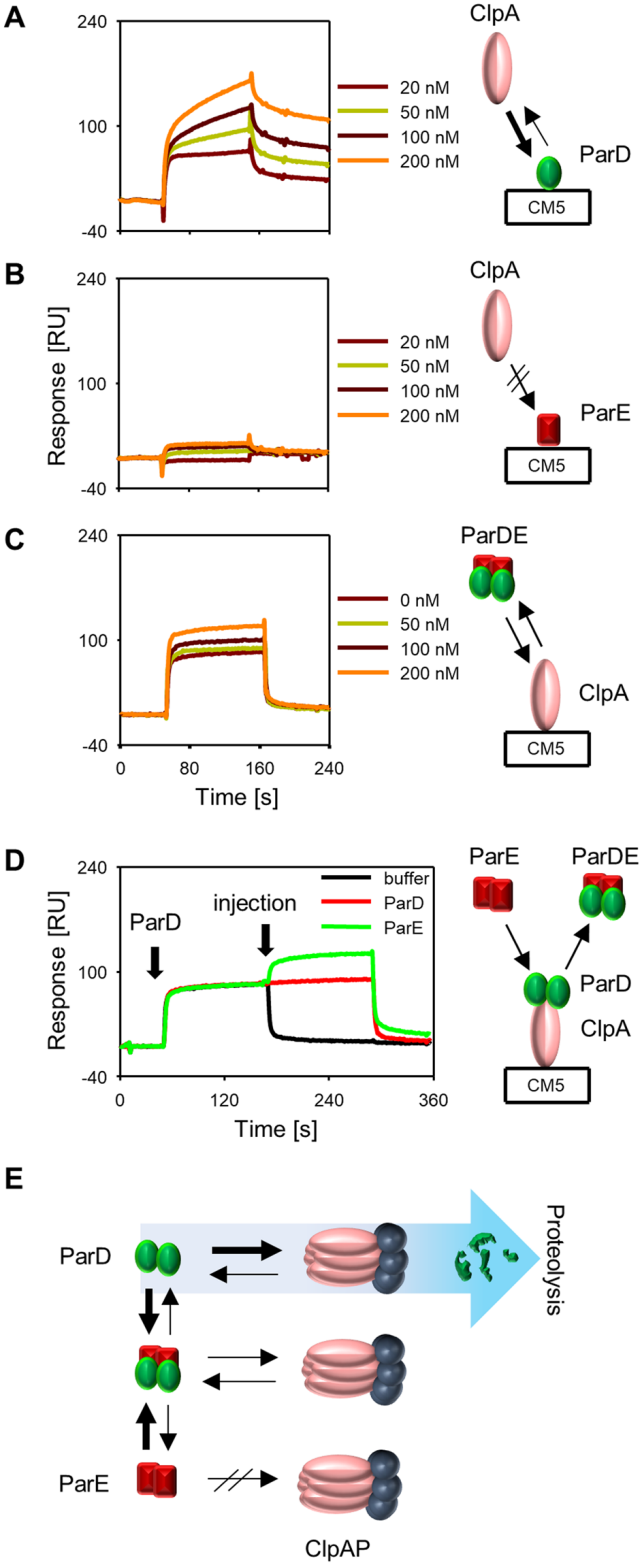
Analysis of ParD and ParE interaction with ClpA by SPR. Experiments were performed as described in Methods. ParD (**A**) and ParE (**B**) were immobilized on the surface of the sensor chip. Indicated concentrations of the ClpA ATPase were injected onto the chip. The buffer was supplemented with 10 mM magnesium acetate and 2 mM ATP. (**C**) ClpA was immobilized on the surface of the chip. Injections of a mixture of ParD (200 nM) protein and increasing concentrations of ParE protein were performed. The buffer was supplemented with 10 mM magnesium acetate and 2 mM ATP. (**D**) ClpA was immobilized on the surface of the chip. Injections of ParD (200 nM) protein were performed to pre-form the ClpA-ParD complex, followed by ParE (200 nM) injection. As a control, ParD or the buffer were injected onto the pre-formed complex. The buffer was supplemented with 10 mM magnesium acetate and 2 mM ATP. (**E**) Schematic representation of ParD, ParE and ClpAP protease interactions. Bold arrows represent a more stable complex.

By utilizing SPR, we performed ClpA, ParD and ParE interaction analysis. The obtained data showed that ClpA, the ATPase unit of ClpAP protease, is responsible for the recognition of the ParD substrate (Fig. 5A), while the ParE toxin is not recognized by ClpA (Fig. 5B). To analyze how the presence of ParE toxin affects the interaction of ParD with ClpA, we injected preincubated ParD and ParE proteins onto ClpA, which was immobilized on a sensor chip. A fixed concentration of 200 nM ParD was incubated with increasing concentrations of ParE (Fig. 5C). The SPR analysis results showed that the potentially created ParD-ParE heterotetamer was able to interact with the ClpA protein. To test whether the pre-formed ClpA-ParD complex might interact with the ParE toxin, we performed an injection of ParE onto the preformed complex of ClpA-ParD on the sensor chip. An increase in response was observed when ParE was injected onto the complex (Fig. 5D). These results indicate that ParE can bind to the ParD-ClpA complex by interacting with ParD. However, if ClpA interacts with another substrate such as TrfA, the ParE toxin does not interact with this complex (see Supplementary Fig. S7). Considering data obtained from SPR and *in vivo* and *in vitro* tests (see Fig. 4) it must be pointed out that formation of ParDE complex does not prevent ParD interaction with ClpA but it protects ParD against proteolysis (Fig. 5E).

### The ClpAP protease is responsible for activating the TA parDE system of the RK2 plasmid in diverse bacterial species

**Figure 6:**
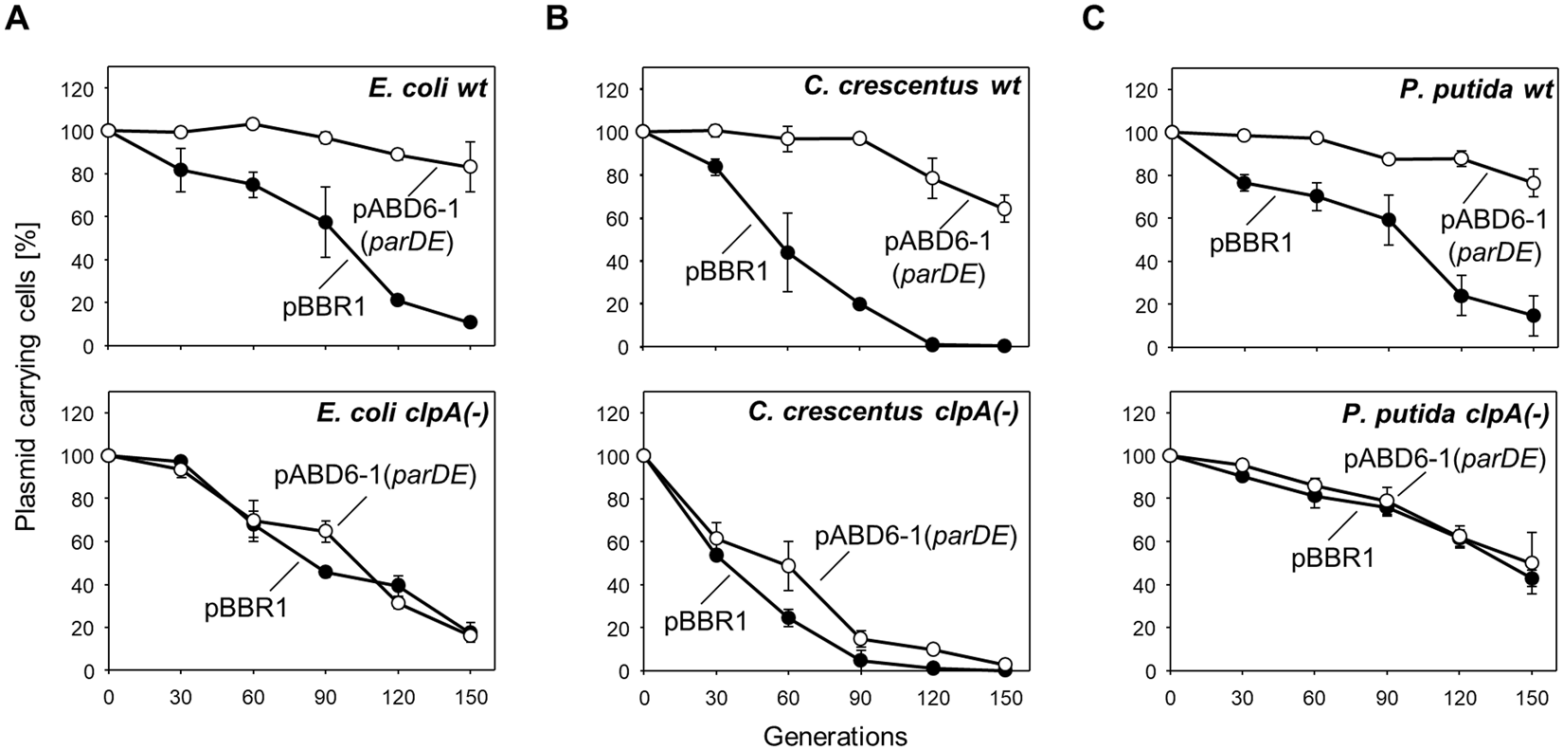
Decreasing stability of the plasmid containing the *parDE* system in *clpA*(−) mutants of various bacterial species. The plasmid stability assay was performed in wild-type (top panels) and *clpA*(−) mutants (bottom panels) of (**A**) *E*. *coli* (**B**) *C*. *crescentus* and (**C**) *P*. *putida* strains carrying pABD6-1 (○), a derivative of pBBR1MSC-5 coding for *parDE* as the only detectable TA gene system, or pBBR1MCS-5 (●) as a control. The experiment was performed as described in Methods. Each experiment was repeated three times, and the mean values with standard deviations (error bars) are presented as graphs. Numeric data and P value are shown in the Supplementary Table S4.

**Figure 7:**
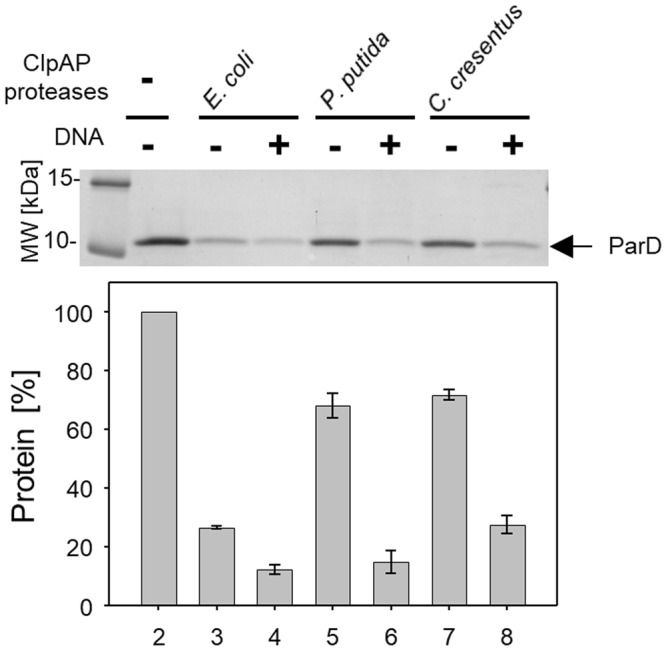
ParD is degraded by ClpAP proteases from different bacterial species. *In vitro* proteolysis assay of ParD antitoxin by ClpAP protease from *E*.*coli* (lanes 3 and 4), *P*. *putida* (lanes 5 and 6) and *C*. *crescentus* (lanes 7 and 8) was performed in the presence (lanes 4, 6 and 8) or absence (3, 5 and 7) of DNA. In a negative control reaction, no protease was added (lane 2). Molecular marker (lane 1). The assay was performed as described in Methods. Full-length gels are included in the Supplementary Information file (Fig. S15). Each experiment was repeated three times, and the mean values with standard deviations (error bars) are presented as graphs.

Plasmid RK2 is present in a number of bacterial species. For this reason we tested whether the ClpAP protease homologs also function as activators of the *parDE* system in other species of bacteria. For this purpose, the operon *parDE* from the RK2 plasmid was cloned into pBBR1MCS-5, a broad host range plasmid lacking any TA system, as described in Methods. Plasmid pABD6-1 (pBBR1-*parDE*) was used to transform wild-type and *clpA*-deficient strains of *E*. *coli*, *P*. *putida*, and *C*. *crescentus*. Plasmid pBBR1MCS-5 was used as a negative control. The maintenance of plasmid pABD6-1 in wild-type *E*. *coli* (Fig. 6A), *C*. *crescentus* (Fig. 6B) and *P*. *putida* (Fig. 6C) cells was on average at the level of 75% after 150 generations compared to the complete loss of the control plasmid. However, in strains with inactive *clpA* genes, no statistically significant difference in the stability of the plasmids was observed (Fig. 6, Supplementary Table S4). These results clearly indicate that the ClpAP protease is responsible for maintaining the plasmid that contains the *parDE* system in different bacterial species. The lack of a functional ClpA unit most likely results in a significant increase in the stability of the ParD antitoxin (as shown in Fig. 1B), thus preventing the toxin activity. We also performed stability tests of plasmids pABD6-1 and pBBR1MCS-5 in the *E*. *coli* strain with inactivation of the *clpX* gene. No differences were observed compared to the wild-type strain (see Supplementary Fig. S8). To analyze the universality of recognition and degradation of the ParD antitoxin by ClpAP, both the ClpA ATPase and the ClpP protease from the bacterial species that host the RK2 plasmid were purified and analyzed for *in vitro* proteolysis (Fig. 7). The results of the *in vitro* proteolytic tests clearly indicated that the ParD antitoxin is degraded by ClpAP proteases from *E*. *coli*, *C*. *crescentus* and *P*. *putida*. Notably, in the case of *C*. *crescentus* and *P*. *putida* proteins, an increase in proteolytic efficiency significantly depends on the presence of DNA in the reaction mixture.

## Discussion

Cellular proteases appear to be the key players activating multiple TA systems via degradation of the proteinaceous antitoxins^45^. Antitoxin stability has been examined in many *E*. *coli* TA systems using *in vivo* analysis, yet little is known about antitoxin stability in other bacteria species. In this work, we have evaluated the degradation of the ParD and ParE proteins of the plasmid RK2 type II TA system by different AAA+ cytosolic proteases of *E*. *coli*, *C*. *crescentus* and *P*. *putida*. The *in vitro* data showed that ClpAP proteases degrade the ParD antitoxin, and *in vivo* tests confirmed these results. In contrast to ParD, the ParE toxin was unaffected by all proteases tested. The results obtained are in agreement with the literature data regarding other type II TA systems^46–48^. This implies a differential stability of ParD and ParE proteins that is the key factor to explain the selective activation of the *parDE* system after plasmid loss.

Our *in vitro* proteolytic assays showed that ParD degradation is rapid; a major reduction of ParD was observed in less than 30 min. We estimated that ParD is degraded by ClpAP with a half-life of approximately 15 min. DNA stimulates this process giving a half-life of approximately 5 min, which is similar to the degradation of the Kis antitoxin by ClpAP^47^. The results of the experiments investigating the order of addition suggest that the observed increase in proteolytic efficiency is the result of direct stimulation of the ClpAP protease by DNA. Our data also show that ClpA ATPase is able to bind to ParD, but not ParE, and the stability of ParD is much higher when it interacts with ParE. Previous data has shown that ParD and ParE create a ParD_2_-ParE_2_ complex^29,34^ where toxicity of ParE is inhibited^36^. When both TA components are present in the host cell, the toxin protects the antitoxin from degradation. The potential cause of the ParD stabilization by the ParE toxin could be the structural change of the ParD antitoxin in complex formation. The structure stabilization of the intrinsically disordered ParD C-terminal due to interaction with ParE has been reported^34^. If the structure change is a reason for preventing ParD protein processing, it must be noted that it does not prevent its recognition by ClpAP. This is evidenced by the results showing that, regardless of whether ParD is associated with ParE, it is recognized by the ClpA subunit. It is not clear if the unstructured part of ParD or a specific motif is recognized by ClpA. The ParD antitoxin sequence alignment with sequences of known ClpA substrate recognition motifs revealed two putative recognition sites, including one motif similar to the *E*. *coli* SsrA recognition sequence. It would be interesting to test whether this is a universal signal for ParD recognition by ClpA homologs. It has been proposed that the N-terminal domain of ClpA facilitates an early binding step, contributing to the specific recognition of substrates for processing^49^. Although the conserved basic residues in the ClpA N-terminal domain were proposed to regulate the processing of several substrates, the molecular mechanism of substrate recognition remains unclear^49^. The mutation of two conserved arginine residues flanking a putative peptide-binding groove within the N-terminal domain of ClpA, specifically compromised the ability of ClpA to unfold and degrade selected substrates, but did not prevent substrate recognition^49^. It is possible that the recognition mechanism is identical among ClpA homologs, which would explain the universality of ParD recognition in various bacterial species. It is also very likely that not all ClpAP substrates would be universally recognized and processed by the homologous ClpAP proteolytic systems, and ParD as a protein of a broad host range replicon evolved as a universal ClpAP substrate. Additional studies on the mechanism of substrate recognition and substrate spectrum are needed to further elucidate this phenomenon.

Our experiments show that the hosts of the RK2 plasmid have a comparable mechanism responsible for the activation of the plasmid *parDE* system. It is interesting that although *C*. *crescentus* has up to four copies of the *parDE* system on the chromosome^50^, the activating factor of each individual systems has not yet been identified. Fiebig and colleagues showed that the expression of the *parDE* operons from *C*. *crescentus* may be dependent on environmental factors such as heavy metals, heat shock, or the culture growth phase^50^. Since RK2 *parDE* stabilizes the plasmid in *C*. *crescentus*, the chromosomal *parDE* systems most likely do not affect the plasmidic TA. However, it is possible that under stress conditions the plasmid could be affected by chromosomal *parDE*. Regarding reports of the *ccdAB* homologous system from *E*. *coli* and the F plasmid^13,14^, it would be very interesting to investigate whether the *parDE* system of the RK2 plasmid may also be involved in the response of the host cells to environmental stress. We observed a slight increase in the stability of ParD in the *E*. *coli lon*(−) strain. There are studies which have described the phenomenon of antitoxin degradation by another protease during environmental stress^51^, although this effect requires further investigation.

The knowledge gained of the TA system that is active in diverse bacterial species might be applied to the development of new strategies for managing pathogens. The plasmid RK2 TA system with its broad host range properties and a universal protease component can be considered as a potential model for the development of new antibacterial strategies.

## Methods

### Bacterial strains, culture conditions, plasmids and oligonucleotides

The *E*. *coli* strains used in this study were as follows: DH5α, BL21(DE3), JM109, and C600 and its derivatives: SG20099 (C600 *clpA::kan*^*R*^), ATC12017 (C600 *lon51*0), SG20080 (C600 *clpX::kan*^*R*^), and SG12065 (C600 *clpY::cat*^*R*^); *P*. *putida* KT2440 and its derivative: AD247((KT2440 *clpA::kan*^*R*^) (this work); and *C*. *cresentus* NA1000 and its derivative: UJ838 (NA1000 *clpA::sm*^*R*^) (U., Jenal). Bacteria were grown in Luria-Bertani (LB) broth with rich medium (RM) (2% peptone, 1% yeast extract, 86 mM NaCl, 0.2% glycerol, 50 mM KPi pH 7.4, 0.1% glucose) or peptone yeast extract (PYE) broth and plated on LB, RM, of PYE with 1.5% agar. Ampicillin (150 μg/ml), kanamycin (50 μg/ml) tetracycline (25 μg/ml), gentamicin (5–15 μg/ml), and spectinomycin (100 μg/ml) were added, as indicated. Plasmids for protein gene expression were: pBAD24-ParD (this work) for expressing *parD* from the arabinose-inducible PBAD promoter, pRR46 (Mini-R6K replicon with the P_parDE_, *parD* and half of *parE*) for constitutive expression of *parD*, pAS12 ((ATG)-*parE* cloned into pGEX-KT) for expression of *parE* from the IPTG-inducible *lac* promotor^29^, pABD2-4 (derivative of pBAD24) for expressing *clpA* from *C*. *crescentus* from the arabinose-inducible PBAD promoter (this work), pET22b-ClpAPpΔHis (derivative of pET22b without C-terminal 6xHis) for expressing *clpA* from *P*. *putida* from the IPTG-inducible *lac* promotor (this work), and pET22b-ClpPPp (derivative of pET22b) for expression of *clpP* from *P*. *putida* from the IPTG-inducible *lac* promotor (this work). The plasmids for the stability assay were pBBR1MCS-5 and pABD6-1 (derivative of pBBR1MCS-5), which has *parDE* as the only detectable TA gene system (this work). Supplementary Table S5 list the primers used in this work.

### DNA manipulations and transformation

Routine DNA recombinant techniques were performed as previously described^52^. Restriction enzymes and other enzymes were used according to the supplier’s instructions. *C*. *crescentus* and *P*. *putida* chromosomal DNA was isolated as described in the A&A Biotechnology Genomic Maxi AX kit. *E*. *coli* transformation was performed using the standard calcium chloride method or by electrotransformation with a Gene-Pulser (Bio-Rad), according to the protocol described by Sambrook *et al*., 1989. Electrotransformation of *C*. *crescentus*, *P*. *putida* and protoplasts with plasmid DNA was performed as previously described^53,54^.

### Protein purification and determination of proteolytic activity

The experiments described in this study utilized highly purified proteins (90% or greater purity). Published protocols were applied for the purification of ParE^29^, ParD^30^, ClpA^55^, ClpP^56^, ClpY^57^, ClpQ^58^, Lon^59^ and TrfA^60^. ClpX was purified using a combination of previously described ion-exchange chromatography methods^61^. The proteolytic activity of the Clp and Lon proteins was measured using a previously described method^44^, with α-casein as a substrate for ClpAP, ClpYQ and Lon, and λO protein for ClpXP. Proteolysis of excess substrate was performed as described for the *in vitro* proteolysis assay. The amount of degraded substrate was estimated after SDS–PAGE, Coomassie staining and densitometry analysis.

### *In vitro* proteolysis assay

Standard proteolysis reactions had a volume of 25 µl and contained 1.5 µg of substrates and 1.5 µg of proteases (Lon (1.5 μg), ClpAP (1.5 μg ClpA and 1.5 μg ClpP), ClpXP (1.5 μg ClpX and 1.5 μg ClpP) or ClpYQ (1.5 μg ClpY and 1.5 μg ClpQ)) in the reaction buffer (40 mM HEPES–KOH pH 7.6, 25 mM Tris–HCl pH 7.6, 4% (w/v) sucrose, 4 mM dithiothreitol, 80 mg/ml BSA, 11 mM magnesium acetate, 4 mM ATP). Additionally, 300 ng of supercoiled plasmid pKD19L1 was included in the reactions containing ParE, ParD, α-casein (see Supplementary Fig. S9A) and λO proteins (see Supplementary Fig. S9B). The reactions were incubated for 2 h at 32 °C, stopped by the addition of 8 µl of 4X Laemmli buffer and analyzed by 12.5% or 15% SDS–PAGE, followed by Coomassie brilliant blue staining. To determine the amount of substrates in each reaction, densitometric analysis was applied using Chemi-Doc Image Lab 5.1 (Bio-Rad) or ImageJ software. All figures were prepared in Microsoft PowerPoint without any modification except for resizing.

### *In vivo* protein stability and Western blot analysis

*In vivo* stability tests were performed in *E*. *coli* C600 and its protease-deficient counterpart, as previously described^61^. These strains were transformed with pRR46 (for constitutive expression of *parD*) and pAS12 or pBAD24-ParD that allows the inducible overexpression of ParE and ParD, respectively. Overnight cultures were diluted 1:100 in fresh LB broth and were grown at 37 °C to an OD_600_ of 0.6. At this point, expression of ParE or ParD was induced. After 1 h of induction, protein synthesis was inhibited by the addition of tetracycline (final concertation of 40 µg/ml) and the samples were collected at indicated time points and suspended in 4X Laemmli buffer. Samples of 15 µl with OD 0.1 were analyzed by 12.5% or 15% SDS–PAGE, followed by Western blotting using anti-ParD (see Supplementary Fig. S10A,B) and anti-ParE (see Supplementary Fig. S10C,D) polyclonal antibodies and a polyclonal goat anti-mouse IgG HRP conjugate. The purified protein was used as a marker. The proteins were visualized by the Chemi-Doc Image Lab 5.1 system (Bio-Rad). All figures were prepared in Microsoft PowerPoint without any modification except for resizing.

### Plasmid stability

The plasmid stability was determined as follows: The tested strains were grown overnight in suitable conditions (27, 30, 32 or 37 °C) in liquid media supplemented with an appropriate antibiotic. Overnight cultures of each strain were diluted 1:100 in fresh LB with antibiotic and grown to an OD_600_ of 0.6. Next, we calculated how many cells were needed to obtain the OD_600_ 0.6 after thirty generations by the equation ${N}_{0}=\frac{{C}_{OD600}}{{2}^{n}}$, where *N*_*0*_ is the initial number of cells, *C*_*OD600*_ is the number of cells in 1 ml of medium with OD_600_ 0.6 and *n* is the number of generations. The culture was then diluted by serial dilution and a proper volume of culture was transferred into 500 ml of fresh, antibiotic-free medium. When the culture reached an OD_600_ of 0.6, the culture was diluted in the same way and left to grow again. Each 30-generation cycle (approximately 12 hours) was repeated until 150 generations of growth under nonselective conditions was reached. Every 30-generation cycle of each culture was plated (approximately 150–200 colonies per plate) on nonselective LB or PYE agar. After overnight incubation, the colonies were examined for resistance to the given antibiotic by replica plating on selective LB or PYE agar. The percentage of plasmid-free cells was estimated from the ratio of antibiotic-sensitive colonies to overall colony number. The percentage of plasmid loss per generation was calculated using the formula $(1-\sqrt[n]{Ft/Fi})\times 100$, as described previously^62^, where *n* the number of generations elapsed, *Fi* is the fraction of cells containing plasmid at the initial time point and *Ft* is the fraction of cells containing plasmid at the final time point.

### Protein interaction analysis

Protein interaction analysis was carried out by applying the surface plasmon resonance (SPR) technique using a BIAcore 2000 and following the manufacturer’s manual, as previously described^55^. The proteins were immobilized on a CM5 Sensor Chip. The running buffer was HBS-EP (150 mM NaCl, 10 mM HEPES pH 7.4, 3 mM EDTA, 0.005% Surfactant P20). The flow rate was set to 15 µl/min, and the volume of injection was 30 µl. The results are presented as sensograms obtained after subtraction of the background response signal from control experiments with buffer injections. The assay was performed in triplicate, and representative sensorgrams are presented. The results were analyzed using BIAevaluation software version 3.2.

### Statistical analysis

To measure whether two sets of data are significantly different from each other we used the two-tailed, homoscedastic T-test. The P values were calculated with using data sets obtained from three repetitions of the analyzed experiments. The P value less than or equal 0.05 was considered statistically significant. The calculations were carried out in the Microsoft Excel software.

## Electronic supplementary material

Supplementary Information
