# Supplementary material for: ClpAP protease is a universal factor that activates the parDE toxin-antitoxin system from a broad host range RK2 plasmid
Source: Sci Rep. 2018 Oct 16;8:15287. doi: 10.1038/s41598-018-33726-y (PMC6191456; doi:10.1038/s41598-018-33726-y)
Supplement: Supplementary file 1 — Supplementary Information [file 41598_2018_33726_MOESM1_ESM.pdf]

SUPPLEMENTART IMFORMATION

ClpAP protease is a universal factor that activates the *parDE* toxin-antitoxin system from a broad host range RK2 plasmid

Andrzej Dubiel[1], Katarzyna Wegrzyn[1], Adam P. Kupinski[1, 2], Igor Konieczny[1]*

[1]Department of Molecular and Cellular Biology, Intercollegiate Faculty of Biotechnology, University of Gdansk and Medical University of Gdansk, Abrahama 58, 80-307 Gdansk, Poland andrzej.dubiel@biotech.ug.edu.pl, katarzyna.wegrzyn@biotech.ug.edu.pl

[2]Current address: Ipsen Bioinnovation, 102 Park Drive, Milton Park, Abingdon, Oxfordshire OX14 4RY, UK, adam.kupinski@ipsen.com

*correspondence: igor.konieczny@biotech.ug.edu.pl

Fig. S1

A

```
ParD      MSRLTIDMTDQQHQSLKALAALQGKTIKQYALERLFPGDADADQAWQELKTMLGNRINDGLAGKVSTKSVGEILDEELSGDRA 83
SsrA      -----------------------AANDENYALAA------------------------------------------------- 11
                                 . . ::***
```

B

```
ParD      MSRLTIDMTDQQHQSLKALAALQGKTIKQYALERLFPGDADADQAWQELKTMLGNRINDGLAGKVSTKSVGEILDEELSGDRA 83
RepA      --------------------------MNQS--------------------------------------FISDILYADIE---- 15
                                    ::*                                         :.:**  ::.
```

Supplementary Figure S1 Alignment of ParD antitoxin sequence with sequence of known ClpA substrate recognition motifs. The sequences alignment was done with Clustal Omega tool (https://www.ebi.ac.uk/Tools/msa/clustalo/). Sequence of ParD antitoxin from RK2 plasmid was aligned with sequence of SsrA motif **(a)** and the 1-15 aa fragment of RepA protein **(b)**, that are known as sequences recognized within a substrate by ClpA protein (Weber-Bann et al., 1999; Hoskins et al., 2000; Hoskins et al., 2002). An * (asterisk) indicates positions which have a single, fully conserved residue; A : (colon) indicates conservation between groups of strongly similar properties; A . (period) indicates conservation between groups of weakly similar properties;

Fig. S2

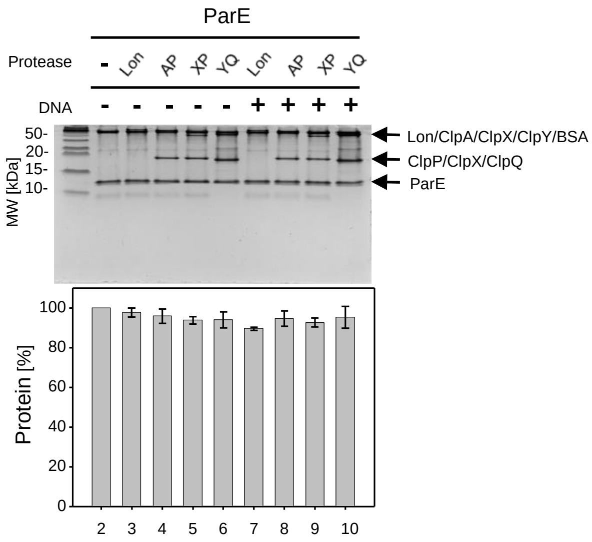

Supplementary Figure S2. *In vitro* proteolysis assay of ParE toxin (1.5 µg) by *E. coli* proteases Lon (lane 3 and 7), ClpAP (lane 4 and 8), ClpXP (lane 5 and 9), and ClpYQ (lane 6 and 10). Supercoiled pKD19L plasmid (300 ng) was added to reaction mixtures (lane 7, 8, 9 and 10). Negative control, no protease added (lane 2). Molecular marker (lane 1). The experiment was carried out for 120 min. The assay was performed as described under Methods.

Fig. S3

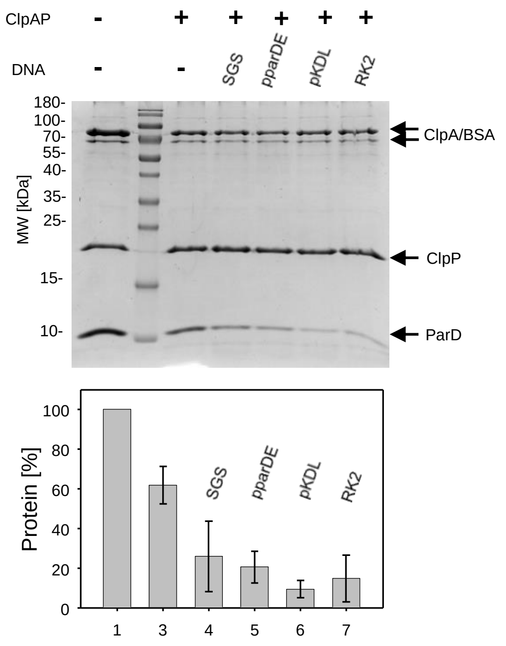

Supplementary Figure S3. Comparison of ParD degradation rate by *E. coli* ClpAP in the presence various DNA. ParD protein (1.5 µg) was incubated with ClpAP and 300 ng of Specific Gyrase Side (SGS) linear DNA (360 bp) (lane 4), promotor fragment of parDE operon (pparDE) linear DNA (250 bp) (lane 5), pKD19-L plasmid supercoiled DNA form (9.1 kbp) (lane 6) and RK2 plasmid supercoiled DNA form (60 kbp) (lane 7). Negative control, reaction was stopped at time 0 (lane 1), reaction control without DNA (lane 3). Molecular weight marker (lane 2). The experiment was carried out for 30 min. The assay was performed as described under Methods. Each experiment was repeated three times, and the mean values with standard deviations (error bars) are presented as graphs.

Fig. S4

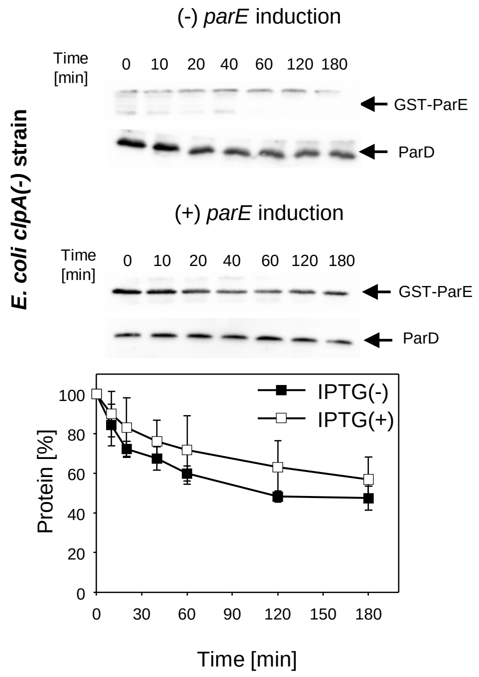

Supplementary Figure S4. *In vivo* stability of ParD in *clpA* deficient strain harboring plasmids for production of ParD (constitutive) and over-production of GST-ParE (IPTG inducible) in the absence (■) or presence (□) of ParE. In both cases, the amount of ParD decrease but the stability is higher than in the wt C600 strain and is independent of ParE. The assay was performed as described under Methods. Samples taken from the cultures at indicated time points analyzed for ParD and ParE presence by SDS–PAGE followed by immuno-blot with anti-ParD and anti-ParE antibodies. Each experiment was repeated three times, and the mean values with standard deviations (error bars) are presented as graphs.

Fig. S5

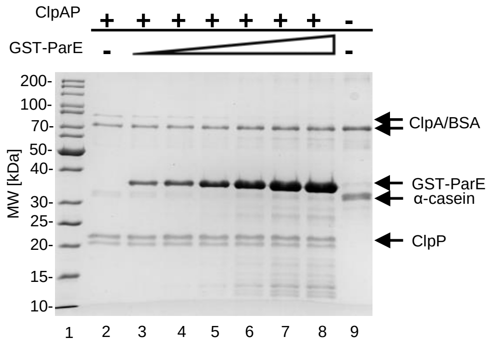

Supplementary Figure S5. α-casein (1.5 µg) *in vitro* proteolysis by ClpAP protease in the presence of ParE. Increasing concentrations of GST-ParE was added to reaction mixtures (lanes 3-8: 0.2, 0.4, 0.75, 1.5, 3 and 6 µg, respectively). In a positive control reaction no GST-ParE was added (lane 2). In a negative control reaction no protease was added (lane 10). Molecular marker (lane 1). The experiment was carried out for 120 min. The assay was performed as described under Methods.

Fig. S6

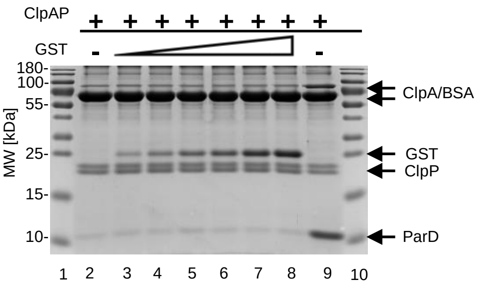

Supplementary Figure S6. ParD (1.5 µg) *in vitro* proteolysis by ClpAP protease in the presence of GST. Increasing concentrations of GST was added to reaction mixtures (lanes 3-8: 0.2, 0.4, 0.75, 1.5, 2 and 3 µg, respectively). In a positive control reaction no GST was added (lane 2). In a negative control reaction was stopped at time 0 (lane 9). Molecular marker (lane 1 and 10). The experiment was carried out for 120 min. The assay was performed as described under Methods.

Fig. S7

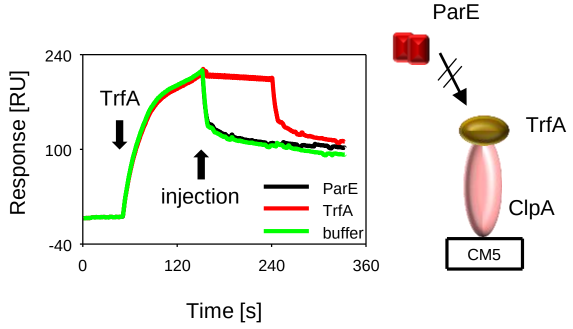

Supplementary Figure S7. Analysis of TrfA interaction with ClpA by SPR. Experiments were performed as described under Methods. ClpA was immobilized on the surface of the chip. Injections of TrfA (200 nM) protein was performed to pre-form ClpA-TrfA complex followed by ParE (200nM) injection. As a control TrfA or the buffer were injected on to the pre-formed complex. The buffer was supplemented with 10 mM magnesium acetate and 2 mM ATP

Fig. S8

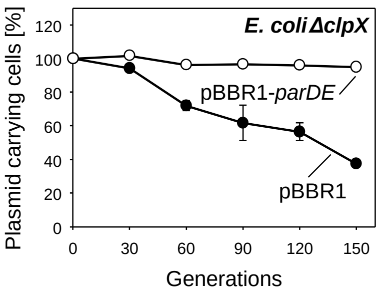

Supplementary Figure S8. Control plasmid stability assay was performed in *E.coli clpX(*-) strains carrying pABD6-1 (○) (derivative of pBBR1MSC-5. coding for *parDE* as the only detectable TA gene system) or pBBR1MCS-5 (●) as a control. Strain was cultured with no antibiotic selection. Experiment was performed as described under Methods. Each experiment was repeated three times, and the mean values with standard deviations (error bars) are presented as graphs.

Fig. S9

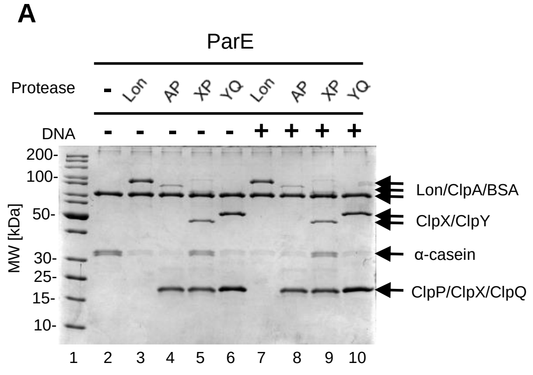

Supplementary Figure S9. Control of proteases activity. **(a)** *In vitro* proteolysis of α-casein by *E. coli* proteases Lon (lane 3 and 7), ClpAP (lane 4 and 8), ClpXP (lane 5 and 9) and ClpYQ (lane 6 and 10). Supercoiled pKD19L plasmid (300 ng) was added to reaction mixtures (lane 7-10). Negative control, no protease added (lane 2). Molecular marker (lane 1). **(b)** *In vitro* proteolysis of λO by *E. coli* proteases ClpXP (lane 3 and 4). Supercoiled pKD19L plasmid (300 ng) was added to reaction mixtures (lane 4). Negative control, no protease added (lane 2). Molecular marker (lane 1). The experiment was carried out for 120 min. The assay was performed as described under Methods.

Fig. S10

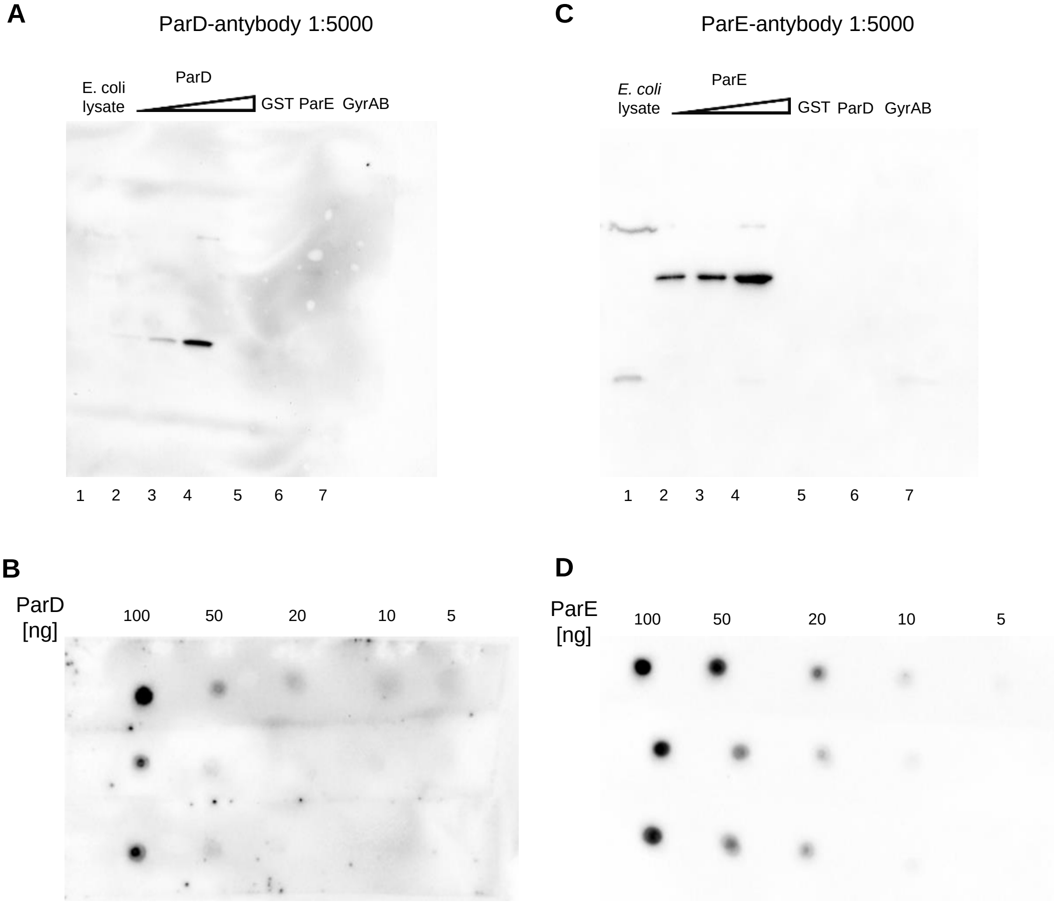

Supplementary Figure S10. Polyclonal antibody control. **(a)** The specificity of anti-ParD antibodies to purified proteins ParD (lane 2-4: 25, 50 and 100 ng respectively) GST (lane 5: 100ng), ParE (lane 6: 100 ng), GyrAB (lane 7: 100 ng) and lysate of *E. coli* C600 (lane 1). Samples of 10 µl in 4X Laemmli buffer were analyzed by 12.5% or 15% SDS–PAGE followed by Western blotting using anti-ParD polyclonal antibody and a polyclonal goat-anti mouse IgG HRP conjugate. **(b)** ParD-antibody titre. Dot-blot of 1 µl sample with indicated amount of protein using anti-ParD polyclonal antibody and a polyclonal goat-anti mouse IgG HRP conjugate. **(c)** The specificity of anti-ParE antibodies to purified proteins ParE (lane 2-4: 25, 50 and 100 ng respectively) GST (lane 5: 100ng), ParD (lane 6: 100 ng), GyrAB (lane 7: 100 ng) and lysate of *E. coli* C600 (lane 1). Samples of 10 µl in 4X Laemmli buffer were analyzed by 12.5% or 15% SDS–PAGE followed by Western blotting using anti-ParE polyclonal antibody and a polyclonal goat-anti mouse IgG HRP conjugate. **(d)** ParE-antibody titre. Dot-blot of 1 µl sample with indicated amount of protein using anti-ParE polyclonal antibody and a polyclonal goat-anti mouse IgG HRP conjugate.

Fig. S11

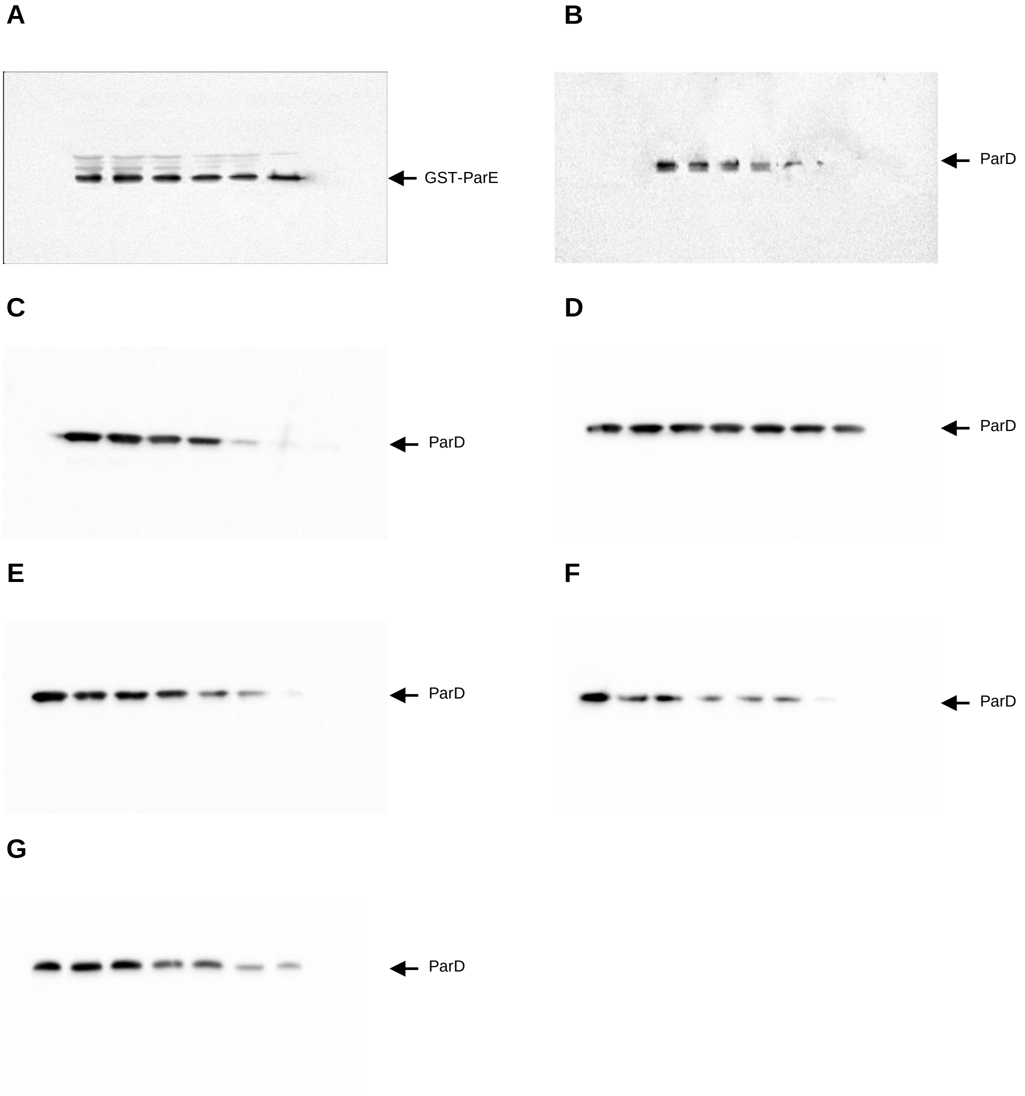

Supplementary Figure S11. Full-length blots shown in (Fig.1). **(a)** ParE stability **(b)** ParD stability and ParD stability in proteases deficient strains of *E. coli* **(c)** *wt*, **(d)** *clpA(-)*, **(e)** *clpX(-)*, **(f)** *clpY(-)* **(g)** *lon(-)*.

Fig. S12

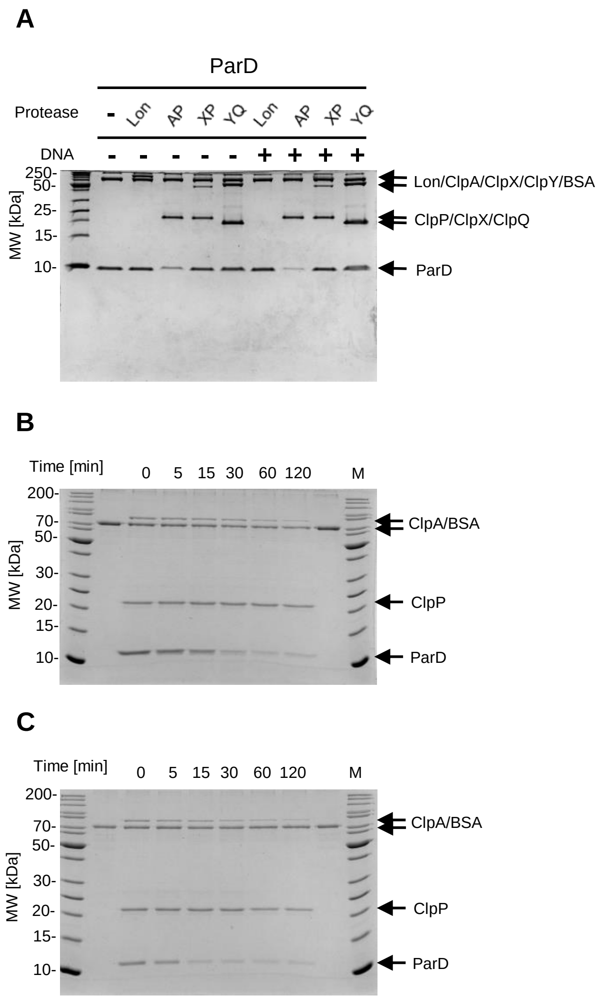

Supplementary Figure S12. Full-length gels shown in (Fig.2). **(a)** *In vitro* proteolysis assay of the analysis of degradation of ParD antitoxin by *E. coli* proteases Lon, ClpAP, ClpXP, and ClpYQ. *In vitro* proteolysis assay of ParD antitoxin by ClpAP **(b)** in the absence or **(c)** presence of DNA.

Fig. S13

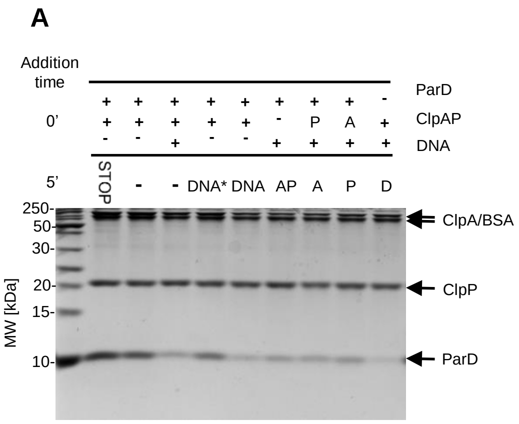

Supplementary Figure S13. Full-length gel shown in (Fig.3). **(a)** *In vitro* proteolysis assay. Reaction components - ParD, pKD19L, pparDE* (linear DNA with parDE promotor sequence) and protease complex subunits (ClpA and ClpP) - were mixed together in various orders.

Fig. S14

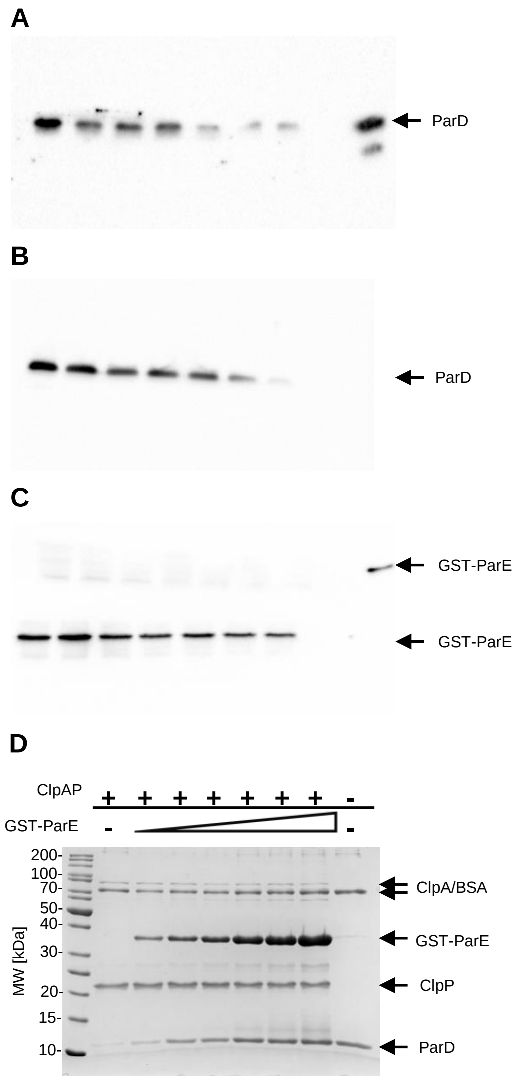

Supplementary Figure S14. Full-length gel and blots shown in (Fig.4) *In vivo* stability of ParD in wild-type strain harboring plasmids for production of ParD (constitutive) and over-production of GST-ParE (IPTG inducible) **(a)** in the absence or **(b)** presence of ParE. **(c)** Control of the ParE presence before induction (top panel IPTG -) and after induction (bottom panel IPTG +). Samples taken from the cultures at indicated time points analyzed for ParD and ParE presence by SDS–PAGE followed by immuno-blot with anti-ParD and anti-ParE antibodies. Purified proteins were used as markers. **(d)** *In vitro* proteolysis assay of ParD antitoxin by ClpAP protease in the presence of ParE.

Fig. S15

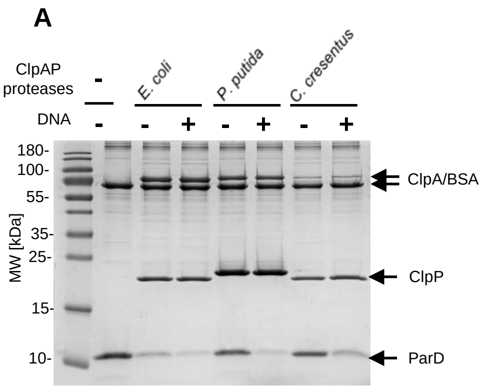

Supplementary Figure S15. Full-length gel shown in (Fig.7) **(a)** *In vitro* proteolysis assay of ParD antitoxin by ClpAP protease from *E. coli*, *P.putida* and *C. crescentus.*

Table S1

Table S1. Numerical values for Fig. 1B. All protease deficient strains were compared to the wt strain

E. Coli strain	Time [min] 0	10	20	40	60	120	180		P value
wt	100	82.2	65.2	57.5	32.6	13.6	1.3	MV [%]	-
	0	8.9	5.2	4.8	20.5	9.0	0.8	SD [%]	
clpA -	100	91.4	84.9	75.6	75.6	77.4	54.6	MV [%]	$1.3x10^{-3}$
	0	6.6	4.7	4.2	8.9	8.3	1.1	SD [%]	
clpX -	100	86.3	85.7	60.3	48.2	33.0	20.2	MV [%]	0.34
	0	10.7	8.2	3.2	13.6	11.4	23.9	SD [%]	
clpY -	100	82.4	64.7	51.6	50.2	37.7	15.1	MV [%]	0.59
	0	10.9	12.8	1.0	3.0	10.9	10.5	SD [%]	
lon -	100	81.8	79.8	45.4	37.4	38.2	25.2	MV [%]	0.56
	0	23.9	17.8	16.8	10.1	10.0	17.9	SD [%]	

Table S2

Table S2. Numerical values for Fig. 2B

DNA presence	Time [min] 0	5	15	30	60	120		V_0 [mol/min]
(-) DNA	100	77.2	54.4	37.4	28.2	23.9	MV [%]	$1.9x10^{-11}$
	0	3.7	5.3	7.5	5.4	3.8	SD [%]	± 0.85
(+) DNA	100	53.4	21.1	16.3	12.3	10.1	MV [%]	$4.9x10^{-12}$
	0	12.5	1.9	3.9	4.2	2.6	SD [%]	± 2.3

P value = $5x10^{-4}$

Table S3

Table S3. Numerical values for Fig. 4A

parE induction	Time [min] 0	10	20	40	60	120	180	
(-) *parE*	100	72	58.4	36.5	12.2	4.1	4.6	MV [%]
	0	13.2	5.1	5.5	5.2	4.1	4.6	SD [%]
(+) *parE*	100	93.7	85.2	69.4	51.5	31.5	8.3	MV [%]
	0	3.6	9.7	1.9	9.8	10.3	6.5	SD [%]

P value = 0.047

Table S4

Table S4. Numerical values for Fig. 6

Plasmid loss per generation [%]

	wt			*clpA* -		
	pBBR1	pABD6-1	P value	pBBR1	pABD6-1	P value
E. coli	1.107 ±0.061	0.129 ±0.095	$7.4x10^{-5}$	1.223 ±0.089	0.886 ±0.19	0.9
C. crescentus	2.991 ±0.372	0.209 ±0.1	$3.7x10^{-5}$	2.812 ±0.324	1.936 ±0.056	0.4
P. putida	1.05 ±0.385	0.178 ±0.055	$1.1x10^{-4}$	0.398 ±0.161	0.369 ±0.051	0.6

Table S5

Table S5. Oligonucleotides used in experiments.

No.	Oligonucleotide sequence	Purpose
1	5’TGTCCCGGGTTAGCGATATAGCG3’	pBAD24-ParD 1
2	5’GGTACCGCGTAAATTGTAAGGGGTTAATATTTTGTTAAAATTGCG TTAAATTTTCGTGTATTTTATGGAG3’	pBAD24-ParD 2
3	5’CGGTCTAGATGCCCTCTTTTTCGCGCC3’	pABD2-4 1
4	5’GGCCAAGCTTCTATTCGGCCATGGCCGG3’	pABD2-4 2
5	5’AAGAAGGAGATATACATATGTTAAACCGCGAGCTCGAAG3’	pET22b-ClpAPpΔHis 1
6	5’GGAGCTCGAATTCGGATCCTCACGCGACCTCAGCCGTG3’	pET22b-ClpAPpΔHis 2
7	5’AAGAAGGAGATATACATATGTCCCGCAATTCTTATATTCAGCAGA GCT3’	pET22b-ClpPPp 1
8	5’GTGGTGGTGGTGCTCGAG GGAGGCCAGTTGCCG3’	pET22b-ClpPPp 2
9	5’CTGTGGATCCTTGGTCAAATTGGGTATAC3’	pABD6-1 1
10	5’TAGAGGATCCTAGCTGTTCTTTGGGTAAGT3’	pABD6-1 2
